# CXCL13 is elevated in inflammatory bowel disease in mice and humans and is implicated in disease pathogenesis

**DOI:** 10.3389/fimmu.2022.997862

**Published:** 2022-09-12

**Authors:** Ting Liu, Yu Liu, Chen-xi Liu, Yong-mei Jiang

**Affiliations:** ^1^ Department of Laboratory Medicine, West China Second University Hospital, and Key Laboratory of Obstetric and Gynecolohic and Pediatric Diseases and Birth Defects of Ministry of Education, Sichuan University, Chengdu, China; ^2^ State Key Laboratory of Biotherapy and Cancer Center/National Collaborative Innovation Center for Biotherapy, Sichuan University, Chengdu, China; ^3^ Department of Laboratory Medicine, West China Hospital, Sichuan University, Chengdu, China

**Keywords:** CXCL13, inflammatory bowel disease, DSS-induced colitis, CXCR5, regulatory B cell

## Abstract

CXCL13 is a chemokine that is widely involved in the pathogenesis of autoimmune diseases, tumors and inflammatory diseases. In this study, we investigate the role of CXCL13 in the pathogenesis of inflammatory bowel disease using both clinical specimens and animal models. We found that the serum CXCL13 concentration in IBD patients was significantly higher than that in healthy controls, and correlated with that of CRP, neutrophils counts and hemoglobin. The increase of CXCL13 in IBD patients might be related to the significant decrease of circulating CD4+CXCR5+ T cells, the increase of CD19+CD5+ B cells and the enhancement of humoral immunity. In mice colitis model, we also found elevated levels of CXCL13 in colon tissue. *Cxcl13^-/-^* knockout mice exhibited a mild, self-limiting form of disease. Additionally, CXCL13 deficiency restricted CD4+CXCR5+ T cells migration in mesenteric lymph nodes, resulting locally regulatory B cells increased in colon. In conclusion, our findings raise the possibility that CXCL13 plays a critical role in the pathogenesis of IBD. We believe that our findings will contribute to the understanding of the etiology, and that antagonizing or inhibiting CXCL13 may work as a potential adjunctive therapy strategy for patients with IBD.

## Introduction

C-X-C motif chemokine ligand 13 (CXCL13), also known as B cell attracting chemokine (BCA-1) or B lymphocyte chemoattractant (BLC), is a kind of chemokine that was originally identified in stromal cells of B cell follicles that regulates the homing of B cells and T cells subsets (1). It is widely involved in the pathogenesis of autoimmunity and a number of inflammatory diseases (1). Accumulated studies have found that in the absence of CXCL13, a reduced inflammatory responses emerges in animal disease models and human pathology studies (2). It is reported that in experimental autoimmune encephalomyelitis (EAE) animal model, the deficiency of CXCL13 results in attenuation of white matter inflammation and gliosis, therefore characterized by a better recovery of disease (3). Consistent with this result, patients with clinically isolated syndrome and multiple sclerosis had high levels of CXCL13 in the cerebrospinal fluid (CSF), and CXCL13 levels are directly related to the number of B cells, T cells and plasmablasts (4–7). For inflammatory diseases caused by infection, studies have found that CXCL13 significantly increased in tuberculosis, HIV, Helicobacter pylori and Clostridium difficile infections (8–12). Therefore, a variety of diseases have been associated with abnormal production of CXCL13 (13–16). Studies have shown that CXCL13 plays an important role in the body’s immune response and inflammatory conditions (17, 18). However, little is known about the contribution of CXCL13 to the development of colon inflammation during inflammatory bowel disease.

Inflammatory bowel disease (IBD) is a chronic, relapsing, lifelong disorder of the gastrointestinal tract, including ulcerative colitis (UC) and Crohn’s disease (CD) two major forms. IBD occurs worldwide and is becoming an important global health problem due to the increase in its incidence noted in the last 50 years. Dextran sulfate sodium (DSS)-induced colitis model is widely used as an animal model of IBD. In the current study, we investigate the role of CXCL13 in the development of both clinical patients with IBD and DSS-induced colitis mice model. We aimed to evaluate the impacts of CXCL13 on inflammatory response, and its feasibility as therapy target for IBD.

## Materials and methods

### Patients

A total of 68 fresh patients newly diagnosed with IBD (without medication treatment) and 34 healthy controls were included in this study. IBD included 34 UC patients and 34 CD patients. Patients as well as healthy controls were recruited from West China Hospital, Sichuan University. The confirmed diagnosis of IBD was based on accepted criteria, including characteristic clinical, endoscopic, radiographic, and histological findings (19). The peripheral blood of patients was collected and the serum was obtained by centrifuge. Informed consent was obtained from all patients before blood analysis. This study was approved by the Medical Ethics Committee of West China Hospital and West China Second University Hospital, Sichuan University.

### Animal

The *Cxcl13^-/-^* knockout mice were purchased from the Jackson Laboratory. The wild-type C57BL/6 mice were purchased from Beijing Vital River Laboratories (Beijing, China), which share the same background as the knockout mice. All the mice were bred locally in the same dedicated pathogen-free facility and co-housed before initiation of DSS colitis. All studies were approved and supervised by the State Key Laboratory of Biotherapy Animal Care and Use Committee (Sichuan University, Chengdu, China).

### Animal experiments

Experimental mice were fed drinking water containing 3.5% dextran sodium sulfate polymers (DSS, MW 36-50 kDa, MP Biomedicals, LLC, Solon, OH, USA) for 5 days, following by distilled water thereafter (n=10/group). As a control, mice in H_2_O group were orally fed with distilled water only. The clinical disease severity (DAI) of all mice in each group was monitored daily, and the specific scoring rules was as follows (20, 21): i) general appearance score rules: normal = 0; piloerection = 1; lethargy and piloerection = 2; motionless, sickly = 4; ii) weight loss score rules: no change = 0; <5% = 1; 6%~10% = 2; 11%~20%= 3; >20% = 4; iii) feces consistency score rules: normal = 0; pasty, semiformed = 2; liquid and sticky = 4; iv) rectal bleeding score rules: no blood = 0; visible blood in the rectum = 2; visible blood on the fur = 4. At the end of the study, spleen and the entire colorectum (from the colorectal junction to the anal verge) were harvested for further analysis. For survival study, 10 mice in *Cxcl13^-/-^* and WT groups were fed with DSS until the day 7 of the experiment, and then returned to distilled water. The survival observation period lasted for 30 days.

### Gross examination and histology

For hematoxylin and eosin (H&E) test, the colon was washed with saline and then immediately fixed with 4% paraformaldehyde in 0.1 M phosphate bufier (pH 7.4). Then, the colon was divided into five equal segments and embedded in paraffin blocks. Paraffin blocks were cut into 3~4 μm thick sections, stained with H&E, and examined with a light microscopy (Leica DFC425C, LAS V3.8 software) at 200× magnification. Histopathological evaluation of the severity of colitis was graded on a scale of 0–4 using a previously validated scoring system (20, 21): i) severity of inflammation: none =0; slight =1; moderate =2; severe =3; ii) mucosal injury depth: none =0; mucosal =1; mucosal and submucosal =2; transmural =3; iii) crypt damage degree: none=0; 1/3 basal damaged =1; 2/3 basal damaged =2; only surface epithelium intact =3; entire crypt and epithelium lost =4; iv) proportion of damage involved: none=0; 1%~25% =1; 26%~50% =2; 51%~75% =3; 76%~100% =4.

### Quantitative real-time PCR

Total RNA was extracted from colon tissues using Axygen RNA extraction kit, and template cDNA was synthesized using the Primescript RT reagent kit (Takara, Tokyo, Japan). The primers are as follow: *Cxcl13*, 5’-TCTCTCCAGGCCACGGTATTCT-3’ (forward) and 5’-ACCATTTGGCACGAGG ATTCAC-3’ (reverse). 18S rRNA, 5’-CGCCGCTAGAGGTGAAATTCT-3’ (forward) and 5’-CGAACCTCCGACTTTCGTTCT-3’ (reverse). All real-time PCR reagents were purchase from Bio-Rad (Bio-Rad, CA, USA), and all reactions were performed on CFX96 real-time system.

### Chemokine and cytokine measurements

Mice colon tissue was weighed and incubated with RIPA buffer containing 1% protease inhibitor cocktail (Sigma-Aldrich, MO, USA) for 2 hours, followed by centrifugation at 17,000g for 20 min at 4°C. Tissue supernatants were collected for Luminex (Merck Millipore, MA, USA) and ELISA test. For human samples, serum CXCL13 and IL-21 levels were measured by ELISA (R&D Systems, CA, USA). C-reactive protein (CRP) and complete blood count analysis was performed using Sysmex-XN9000 (Sysmex Corporation, Kobe, Japan).

### Flow cytometry

The anti-mouse antibodies (BD Biosciences and BioLegend) used for flow cytometry analysis included: PE conjugated anti-CD4, PerCP conjugated anti-B220, APC conjugated anti-CD5 and APC conjugated anti-CXCR5. The anti-Human antibodies (BD Biosciences and BioLegend) used for flow cytometry analysis included: APC-cy7 conjugated anti-CD45, PerCP conjugated anti-CD3, FITC conjugated anti-CD4, PE conjugated anti-CD8, AmCyan conjugated anti-CD19, APC conjugated anti-CD5 and Pacific blue conjugatedanti-CXCR5. Cells were co-incubated with antibodies for 30 minutes in dark at 4°C and then analyzed with BD FACS Canto II (BD Biosciences, CA, USA). Data was analyzed by Kaluza Flow Cytometry Analysis Software.

### Statistical analysis

The experimental data was statistically analyzed by SPSS22.0 software. Sample size was calculated by Power and Sample Size software (Type I error rate=0.05, Power=0.9). All data were expressed as mean ± SEM. The data comparison between the two groups was performed by two-tailed Student’s t test, and *P*<0.05 indicated that the difference was statistically significant.

## Results

### Serum CXCL13 levels are correlated with inflammatory response in clinical IBD patients

**Figure 1:**
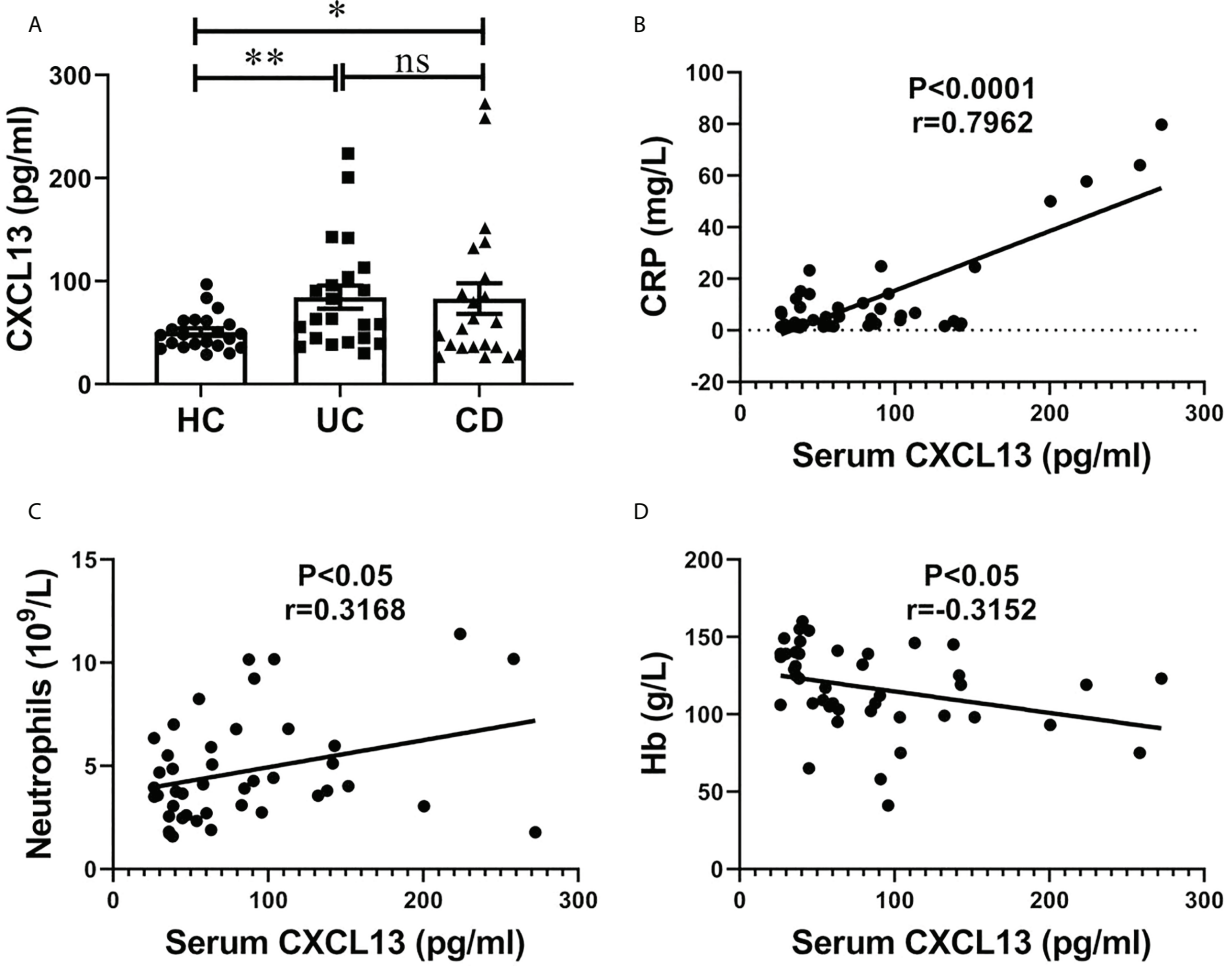
Serum CXCL13 level was correlated with the severity of inflammatory response in patients with IBD. **(A)** ELISA analysis of CXCL13 in serum of IBD patients and healthy controls (n=22/group). Data are presented as mean ± SEM, with *P* values determined by two-tailed Student’s t test. **(B–D)** Correlation between serum CXCL13 levels and C-reactive protein, neutrophils counts, hemoglobin were assessed in clinical IBD patients. Statistically significant correlations were determined using linear regression. These experimental data indicate that CXCL13 is significantly elevated in the serum of IBD patients, and its expression is positively correlated with the degree of inflammatory response. *P <0.05; **P < 0.01. ns, not statistically significant.

The serum CXCL13 levels in IBD patients and healthy controls are shown in **Figure 1**. Significantly higher serum CXCL13 level was observed in both UC and CD patients compared with healthy control (**Figure 1A**, n=22/group). We next assessed whether inflammation marker C-reactive protein (CRP) and neutrophils counts, and anemia marker hemoglobin content (Hb) were correlated with serum CXCL13 levels in the IBD patients. Interestingly, we observed a correlation between CXCL13 and inflammation severity. We found a positive correlation of CXCL13 with CRP and neutrophils counts, while a negative correlation of CXCL13 with Hb (**Figures 1B–D**). For CRP and neutrophils, they are serum indicators of inflammatory response, thus they are positively correlated with CXCL13. For Hb, since the main symptoms of IBD patients are diarrhea and rectal bleeding, patients have reduced hemoglobin in peripheral blood. Therefore, Hb is negatively correlated with disease severity, and ultimately negatively correlated with CXCL13. All the above results indicated that serum CXCL13 level are involved in the regulation of inflammatory response during IBD development.

### CXCL13 regulates peripheral blood CD4+CXCR5+ T cells and CD19+CD5+ B cells, thereby promoting IgA production in IBD patients

**Figure 2:**
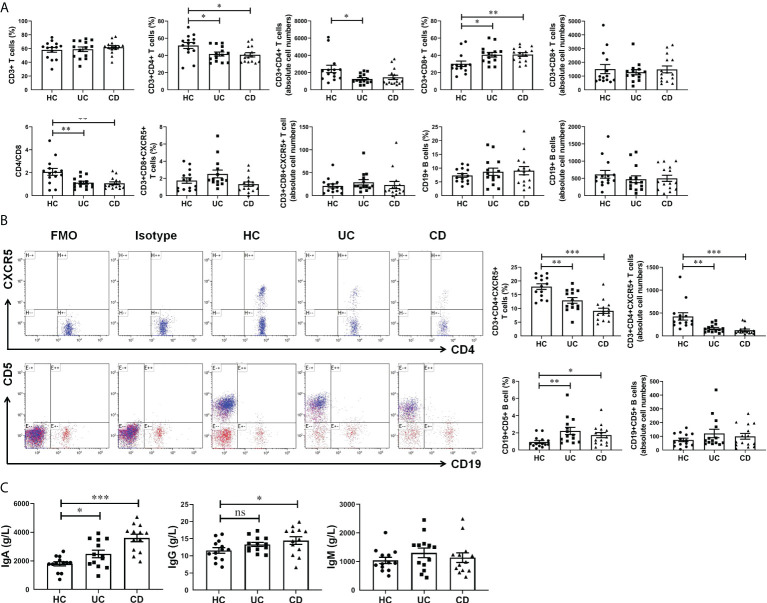
CXCL13 regulated CD4+CXCR5+ T cells and CD19+CD5+ B cells in IBD patients, and therefore enhanced antibody secretion. **(A, B)** Flow cytometry analysis of T cells and B cells subsets in peripheral blood of IBD patients and healthy controls. **(C)** Electrochemiluminescence analysis of IgA, IgG and IgM in serum of IBD patients and healthy controls. The results reflected the cellular and humoral immunity of IBD patients. These results indicate that during the course of IBD patients, CD4+CXCR5+ T cells decreased, CD19+CD5+ B cells increased, and IgA antibody secretion increased. This phenomenon may be resulted from the elevation of CXCL13 in IBD patients. Summary data are presented as mean ± SEM (n=13-15/group), with *P* values determined by two-tailed Student’s t test. *P <0.05; **P < 0.01; ***P < 0.001. ns, not statistically significant.

For cell-mediated immunity, we found no significant difference in both percentage and absolute cell numbers of CD45+CD3+ T cells and CD45+CD19+ B cells between IBD patients and healthy controls. The percentage of CD45+CD3+CD4+ T cells were significantly decreased in UC and CD groups, while CD45+CD3+CD8+ T cells and CD45+CD19+CD5+ B cells were significantly increased (**Figures 2A, B**). In addition, there was no statistically significant difference in the percentage of CD3+CD8+CXCR5+ T cells among the three groups. While the percentage of CD4+CXCR5+ T cells in CD45+CD3+ T cells of peripheral blood were significantly reduced in patients with IBD, which we speculated might be related to the increase of the serum chemokine CXCL13. Notably, the absolute cell number of CD4+CXCR5+ T cells was significantly reduced in IBD patients, suggesting that the observed percentage changes were due to the alterations of CD4+CXCR5+ T cells themselves (**Figure 2B**). In terms of humoral immunity, IgA was significantly increased in the serum of patients with UC and more in patients with CD (**Figure 2C**). IgG also increased in the serum of UC and CD patients, and the difference between HC and CD was statistically significant. It is worth mentioning that IgM increased to a lesser extent in patients with IBD, which may be related to the selection of disease course. Therefore, in peripheral blood circulation of patients newly diagnosed with IBD, CD4+CXCR5+ T cells decreased, CD19+CD5+ B cells increased, and IgA antibody secretion increased. These results may be related to the elevation of CXCL13.

### CXCL13 deficiency alleviates the inflammatory response of DSS-induced colitis in mice

**Figure 3:**
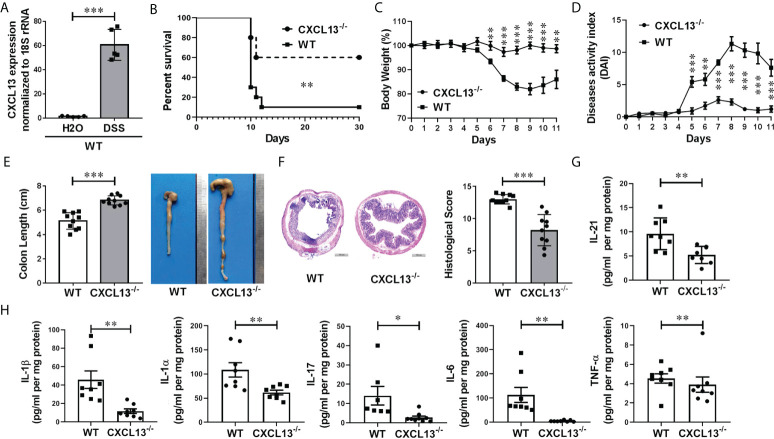
CXCL13 deficiency inhibits the occurrence and development of DSS-induced colitis. **(A)** Real-time PCR analysis of CXCL13 mRNA level in colon tissue of colitis and healthy mice (n=5/group). **(B)** Survival curve of WT and *Cxcl13^-/-^* mice treated with 3.5% DSS drinking water for 7 days and then fed with regular drinking water for the rest of time (n=10/group). **(C–F)** Body weight changes **(C)**, diseases activity index **(D)**, colon lengths **(E)**, H&E-stained colon sections and histology scores **(F)** of WT and *Cxcl13^-/-^* mice treated with 3.5% DSS drinking water for 5 days and then fed with regular drinking water for 6 days (n=10/group). **(G, H)** ELISA and Luminex analysis of tissue cytokines on day 11 of WT or *Cxcl13^-/^* mice treated with 3.5% DSS drinking water for 5 days and then fed with regular drinking water for 6 days (n=8/group). These results suggest that CXCL13 deficiency can effectively alleviate the severity of DSS-induced colitis, including improved survival, relieved weight loss and disease activity, and an effective reduction in inflammatory infiltration at the site of inflammatory the colon. Summary data are presented as mean ± SEM based on multiple mice, with *P* values determined by Kaplan-Meier analyses with the log-rank Mantel-Cox test **(B)**, or two-tailed Student’s t test **(C–H)**. *P <0.05; **P < 0.01; ***P < 0.001.

To investigate whether CXCL13 regulates the inflammatory response in IBD, we employed a classic DSS-induced experimental colitis model. With C57BL/6 mice, we detected CXCL13 expression in the colorectal region of colitis and healthy mice using real-time PCR. Interestingly, DSS treatment resulted in a significant increase in the level of CXCL13 in colon tissue, suggesting increase expression of CXCL13 at site of intestinal inflammation along with DSS-induced colitis (**Figure 3A**). To determine the pathogenic mechanism of CXCL13, we treated the wild-type (WT) and *Cxcl13^-/-^* knockout mice with 3.5% DSS in drinking water. Of the *Cxcl13^-/-^* mice, 60% survived the treatment up to 30 days, but only 10% of WT mice survived under the same conditions (**Figure 3B**). The *Cxcl13^-/-^* mice also displayed less severe body weight loss and lower disease activity index scores than WT mice (**Figures 3C, D**). Consistently, the *Cxcl13^-/-^* mice experienced less colon shortening, which was the characteristic of colitis (**Figure 3E**). Histology analysis showed that distal colon inflammation and tissue damage were significantly more severe in WT mice than in *Cxcl13^-/-^* mice (**Figure 3F**). Quantitative analysis also revealed significantly lower histological colitis scores in the colon of *Cxcl13^-/-^* mice (**Figure 3F**). We next examined the effect of CXCL13 deficiency on pro-inflammatory cytokines secretion during DSS-mediated colitis. Compared to WT mice, the *Cxcl13^-/-^* mice had significantly decreased concentration of several pro-inflammatory cytokines, including IL-21, IL-1β, IL-1α, IL-17, IL-6 and TNF-α on day 11 of DSS treatment (**Figures 3G, H**). Thus, these results indicated that CXCL13 plays a crucial role in exacerbating the occurrence and development of colonic inflammation in mice colitis model.

### CXCL13 deficiency restricts the migration of CD4+CXCR5+ T cells in mesenteric lymph nodes, thereby promoting the increasing of regulatory B cells in colon

**Figure 4:**
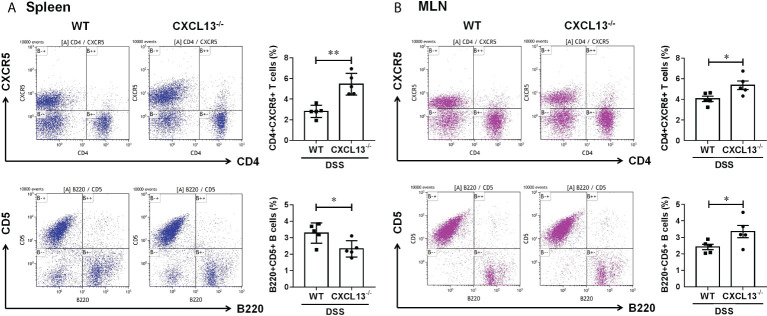
CXCL13 regulates the recruitment of CD4+CXCR5+ T cells in colitis mice, thereby promoting the increasing of regulatory B cells in colon. Flow cytometry analysis of CD4+CXCR5+ T cells and B220+CD5+ B cells in **(A)** spleen and **(B)** MLN of WT and *Cxcl13^-/-^* mice with colitis. In *Cxcl13^-/-^* knockout mice, CD4+CXCR5+ T cells were significantly increased in both spleen and MLN, while CD5+ B cells were decreased in spleen and increased in MLN. These data suggest that CXCL13 is involved in inhibiting CD4+CXCR5+ T cell recruitment to secondary lymphoid organs from circulating and MLN. The increase of CD4+CXCR5+ T cells in MLN of *Cxcl13^-/-^* mice promoted the increase of CD5+B cells in local colon tissue. Summary data are presented as mean ± SEM based on multiple mice, with *P* values determined by two-tailed Student’s t test. *P <0.05; **P < 0.01.

On day 11 of DSS-induced colitis, spleen and mesenteric lymph nodes (MLN) of mice were taken to detect the percentage of CD4+CXCR5+ T cells and regulatory CD5+B cells by flow cytometry. It was found that the CD4+CXCR5+ T cells in both spleen (**Figure 4A**) and MLN (**Figure 4B**) of *Cxcl13^-/-^* mice were significantly increased, while the CD5+ B cells were decreased in spleen and increased in MLN. The results suggest that CXCL13 is involved in inhibiting CD4+CXCR5+ T cell recruitment to secondary lymphoid organs from circulating and MLN. The increase of CD4+CXCR5+ T cells in MLN of *Cxcl13^-/-^* mice promoted the maturation of B cells, suggesting the critical role of CXCL13 in regulating humoral immunity in colon.

## Discussion

In this study, we have demonstrated that the serum CXCL13 levels were markedly increased in both IBD patients (**Figure 1A**) and mice colitis models (**Figure 3A**), and its expression level was positively correlated with the severity of inflammatory response (**Figures 1B–D**). Moreover, there was no significant difference in the percentage of CD3+ T cells and CD19+ B cells in peripheral blood among patients with ulcerative colitis, crohn’s disease, and healthy controls (**Figure 2A**). The percentage of CD3+CD4+ T cells, CD45+CD3+CD4+CXCR5+ T cells and CD4/CD8 ratio in peripheral blood of patients with ulcerative colitis and crohn’s disease was significantly lower than that of healthy controls, while the percentage of CD3+CD8+ T cells and CD19+CD5+ B cells in peripheral blood of patients with ulcerative colitis and crohn’s disease was significantly higher than that of healthy controls (**Figures 2A, B**). We speculated that the cause of this result might be the increase of serum CXCL13 in IBD patients, which promoted CD4+CXCR5+ T cells homing to the follicular area of lymph nodes, significantly decreased the percentage of CD4+CXCR5+ T cells in peripheral blood circulation, and eventually lead to an increase in inhibitory CD19+CD5+ B cells and thereby enhancing humoral immunity (**Figure 2C**). However, some previous studies had shown that CXCR5+ T follicular helper (Tfh) cells participate in the pathogenesis of IBD, and its level was elevated in UC patients (22, 23). Such inconsistencies may be related to the differences in the course of disease among the participants. In our study, the selected patients were all newly diagnosed IBD patients at the early stage of the disease. In addition, CD4+CXCR5+ T cells included not only CD4+CXCR5+Foxp3- T follicular helper (Tfh) cells, but also CD4+CXCR5+Foxp3+ T follicular regulator (Tfr) cells (24, 25). Furthermore, *Cxcl13^-/-^* knockout mice were used to construct DSS-induced colitis model to verify the role of CXCL13 in IBD. Compared with WT mice, *Cxcl13^-/-^* knockout mice had less symptoms of colitis, including longer survival, less weight loss, less disease activity, less colon length shorten, and less colorectal inflammatory infiltration (**Figures 3B–F**). The deficiency of CXCL13 resulted in significantly decreased expression of inflammatory cytokines IL-21, IL-1β, IL-1α, IL-17, IL-6 and TNF-α in colon sites of mice with colitis (**Figures 3G, H**). The expression level of cytokines in colon tissue of mice was consistent with its clinical manifestations. In terms of cellular immunity, CD4+CXCR5+ T cells were significantly increased in spleen and mesenteric lymph nodes, suggesting that CXCL13 reduced the recruitment of CD4+CXCR5+ T cells to secondary lymphoid organs, and restricted CD4+CXCR5+ T cells to circulating and mesenteric lymph nodes (**Figure 3A**). Most studies considered T follicular helper cells as the key cells in regulating germinal center formation and B cell function (1, 26). Our study found that reduced recruitment of CXCR5+ T cells to secondary lymphoid organs caused by CXCL13 deficiency resulted in an increase of CXCR5+ T cells and a decrease of regulatory CD5+ B cells in circulating, while the increase of CXCR5+ T cells in mesenteric lymph nodes resulted in a significant increase of regulatory CD5+ B cells at the site of intestinal inflammation. This could explain why CXCL13 deficiency can effectively alleviate DSS-induced colitis in mice. In summary, the clinical results of this study are consistent with those of animal experiments.

In conclusion, our study demonstrated a elevation of CXCL13 in both IBD patients and DSS-induced colitis mice, which contributed to the recruitment of CD4+CXCR5+ T cells to secondary lymphoid organs, thereby promoting the production of regulatory CD5+ B cells. Furthermore, CXCL13 is involved in the pathogenesis of inflammatory bowel disease, and the concentration of CXCL13 is positively correlated with the severity of the inflammatory response. The deficiency of CXCL13 can effectively alleviate the occurrence and the development of DSS-induced colitis by limiting CD4+CXCR5+ T cells migration from mesenteric lymph nodes to secondary lymphoid organs, and thereby inducing local regulatory B cells increase in colon. The increase of regulatory B cells in mesenteric lymph nodes can further promote the secretion of mucosal antibodies and enhance humoral immunity. Thus, chemokine CXCL13 might pose a risk factor affecting the inflammatory process of IBD, and it plays an important role in the regulation of cellular and humoral immunity. Our study may provide new targets and therapeutic strategies for the immunotherapy of IBD.

Our study still had several limitations. The trial involved only a limited number of clinical participants, therefore we may need a larger sample size to further validate our proposed finding. Moreover, we have studied the regulation of CXCL13 on CD4+CXCR5+ T cells and CD5+ B cells, but the specific molecular mechanism need to be further studied. Finally, the effect of CXCL13 on intestinal microecology and its underlying mechanism also need to be further studied.

## Data availability statement

The original contributions presented in the study are included in the article/supplementary material. Further inquiries can be directed to the corresponding authors.

## Ethics statement

The studies involving human participants were reviewed and approved by Medical Ethics Committee of West China Hospital and West China Second University Hospital, Sichuan University. The patients/participants provided their written informed consent to participate in this study. The animal study was reviewed and approved by State Key Laboratory of Biotherapy Animal Care and Use Committee.

## Author contributions

TL drafted the manuscript. TL, YL, and C-xL generated the experimental results. Y-mJ reviewed the manuscript for intellectual content. All authors approved the final version of the manuscript. All the authors have accepted responsibility for the entire content of this submitted manuscript and approved submission.

## Funding

This study was generously supported by a grant from the National Natural Science Foundation of China (No. 81801628) and the Clinical Research Foundation of West China Second University Hospital (No. KL076). We express our great thanks to them.

## Acknowledgments

We sincerely thank Prof. Yu-Quan Wei, the director and professor in State Key Laboratory of Biotherapy and Collaborative Innovation Center for Biotherapy, Sichuan University, Chengdu, China, for his continued support and encouragement.

## Conflict of interest

The authors declare that the research was conducted in the absence of any commercial or financial relationships that could be construed as a potential conflict of interest.

## Publisher’s note

All claims expressed in this article are solely those of the authors and do not necessarily represent those of their affiliated organizations, or those of the publisher, the editors and the reviewers. Any product that may be evaluated in this article, or claim that may be made by its manufacturer, is not guaranteed or endorsed by the publisher.

## Abbreviations

CXCL13, C-X-C motif chemokine ligand 13; BCA-1, B cell attracting chemokine; BLC, B lymphocyte chemoattractant; EAE, experimental autoimmune encephalomyelitis; CSF, cerebrospinal fluid; IBD, inflammatory bowel disease; UC, ulcerative colitis; CD, Crohn’s disease; DSS, dextran sodium sulfate polymers; DAI, clinical disease severity; H&E, hematoxylin and eosin; CRP, C-reactive protein; Hb, hemoglobin; WT, wild-type; MLN, mesenteric lymph nodes; Tfh, T follicular helper cells; Tfr, T follicular regulator cells.
